# Psychosocial Implications During Adolescence for Infant Heart Transplant Recipients

**DOI:** 10.2174/157340311797484277

**Published:** 2011-05

**Authors:** Vidhya Krishnamurthy, Catherin Freier Randall, Richard Chinnock

**Affiliations:** Loma Linda University Children’s Hospital Loma Linda, CA

**Keywords:** Cognition, Neuropsychology, Pediatric Heart Transplant, Psychosocial, Quality of Life, Self-Esteem, Social Skills.

## Abstract

**Background & Objectives:**

As more heart transplant recipients survive into late adolescence, research addressing long-term psychosocial and neurodevelopmental outcomes is imperative. The limited literature available suggests risk for psychosocial difficulties and lower cognitive, academic, and neuropsychological functioning. This paper reviews topic-related literature and provides preliminary data examining psychosocial and neuropsychological functioning of adolescents who received their heart transplant during infancy.

**Method:**

This paper offers a literature review AND presents preliminary data from studies conducted through Loma Linda University Children’s Hospital (LLUCH). Study one examined psychosocial functioning and quality of life of adolescent infant heart transplant recipients. In study two, cognitive, academic, and neuropsychological data were analyzed.

**Results:**

**Study 1:** Overall psychosocial functioning fell in the Average range, however, a significant percentage of participants presented with difficulties on one or more of the psychosocial domains. Quality of life was also within normal limits, though concerns with general health and bodily discomfort were noted. **Study 2:** Cognitive functioning was assessed to be Below Average, with 43-62% of the participants demonstrating significant impairments. Neuropsychological functioning yielded significant weakness on language functioning, and mild weakness on visual-motor integration and executive functioning.

**Conclusion:**

While the majority of the participants demonstrate psychosocial resiliency, a subgroup present with difficulties suggesting the need for intervention. Cognitive/neuropsychological functioning suggests poorer functioning with patterns similar to other high-risk pediatric populations. These results are preliminary and further research on long-term psychosocial and neuropsychological development of pediatric heart transplant recipients is needed to better understand and ameliorate developmental trajectories.

## INTRODUCTION

In order to best understand the psychosocial considerations for the adolescent who has been a recipient of a heart transplant it is important to note the primary tasks of typical adolescence. Adolescence has generally been considered to be a transitional period from childhood to adulthood. In recent decades, however, considerable research has focused on this period due to the number of distinctive changes that is noted to occur during this time. Researchers have focused on three primary forms of changes – biological, social, and cognitive [1]. Significant physical changes associated with puberty marks the beginning of the period. Transformations in parent-child and peer relationships as well as increase in the social contexts that adolescents engage in are also hallmarks of this period. Literature has shown that the relationship between parent-child alters considerably as adolescents attempt to gain autonomy and transition in adulthood [1,3-4]. Further, individuals are noted to spend considerably more time with peers and place more importance on peer relationships during adolescence than childhood [2]. Intimate romantic relationships also begin to emerge during this period. The last form of change noted during the adolescent period is in their cognitive functioning as abstract reasoning and critical thinking skills further develop. The interaction of these forms of change and environmental and societal context within which an individual exists is noted to produce the developmental outcomes of this period – adaptive psychosocial functioning, self-identity, and autonomy [1]. 

Given the significant developmental changes that occur during adolescence, this period is frequently depicted as a time of stress and conflict [1]. Grotevant explains that the changing parent-child and peer relationships, the choices and decisions frequently confronting them, and the expectations placed on them can cause typical adolescents to experience significant levels of stress and anxiety from time to time. This raises the question of the emotional and behavioral status of adolescents who are experiencing significant biological and environmental stressors, such as those with a chronic medical condition. Adolescents with chronic illnesses face unique challenges in their attempts to gain autonomy and transform their relationships with their parents or caregivers [5]. They may also experience social delays due to their medical history and difficulty in forming age-appropriate peer relationships [5]. Additionally, they can evidence cognitive deficits that prevent them from acquiring the skills that are noted to emerge during adolescence [6-9]. This underscores the potential for psychosocial difficulties and identity problems in adolescents with chronic medical conditions that need to be explored further. In line with the intent of this paper, these issues will be addressed in adolescents who are recipients of a heart transplant.

### Pediatric Heart Transplantation

Since 1982, more than 8500 pediatric heart transplantations have been successfully completed around the world [10]. Of these 8500, 24% were infant heart transplantations. The median survival time period for pediatric transplantation was 11.3 years and infant transplantation was 18.3 years. This data from the International Heart and Lung Transplantation (ISHLT) registry indicates that the majority of the transplant recipients are surviving into their late adolescence and early adulthood. Given the improved health outcomes of the pediatric transplant recipients, research attention has begun to focus on growth, development (cognitive and psychosocial), and quality of life of the adolescent who has been a heart transplant recipient. 

### Purpose

The purpose of this paper is two fold; first, to review the literature regarding current understanding and findings on the psychosocial and neurodevelopmental functioning of adolescents who have undergone a heart transplant. Second, we provide preliminary descriptive data from several studies being conducted at Loma Linda University International Heart Institute with adolescents who underwent heart transplantation prior to the age of one year. This is a very unique population in that they have had to live with the transplantation and treatment follow-up almost since birth unlike the majority of current adolescent transplantation patients who would have generally acquired the need for transplantation later on in life. Further, since Loma Linda University performed the first successful newborn heart transplant it represents the oldest infant heart transplant recipients nationally. In keeping with the articles’ intent, we will examine in our first study the psychosocial functioning and quality of life of infant heart transplant recipients during adolescence. In the second study we discuss neurodevelopmental outcomes and provide preliminary cognitive and neuropsychological data obtained from older adolescents and young adults. 

## STUDY 1: PSYCHOSOCIAL FUNCTIONING IN ADOLESCENTS WHO ARE PEDIATRIC HEART TRANSPLANT RECIPIENTS

Recipients are frequently noted to experience significant improvements in functioning after the transplant as they experience fewer hospitalizations and are able to engage in age-appropriate activities, such as attending schools, participating in sports, and spending time with friends [5,11]. Recipients, however, also need to be on immunosuppressant therapy life-long to prevent organ rejection and cope with dietary and activity limitations [5,12]. They still need to attend regular clinical appointments to monitor functioning and undergo laboratory tests and medical procedures. Further, the immunosuppressant therapy is noted to have adverse side effects in that it alters the physical appearance of users, may cause mood fluctuations and cognitive changes, and increases the risk of infections [12]. Additionally, recipients constantly face the fear of organ rejection and future health status concerns [5]. Given the chronic health stressors that recipients experience, there are concerns noted in the literature regarding their psychosocial functioning and potential for developing an Affective Disorder, such as Anxiety, Depresssion, or Posttraumatic Stress Disorder (PTSD) [11,13-19]. 

### Social Functioning

As mentioned previously, peer relationship becomes increasingly more important as children grow into adolescence. As such, age-appropriate social experiences and skills are necessary to develop those relationships. While studies examining the social functioning of heart transplant recipients are limited those that focus on adolescent transplant recipients are virtually nonexistent. The few studies that have examined the social functioning of children and adolescent heart transplant recipients have consistently found that recipients tend to have poorer social skills than their healthy peers [5,11,20]. Uzark *et al* (1992) found that transplant recipients were overall assessed to have lower social competence than their healthier counterparts, and that over one third of them were below normal range [11]. Wray and Radley-Smith (2006) found that over half of the pediatric heart transplant recipients in their study were assessed to have significant social deficits, with boys, in particular, obtaning very low scores on this domain [20]. 

The lower social competence evidenced by the recipients has generally been attributed to their absence from normal social situations that would promote social skills, such as school, playdates, and extracurricular activities [5,11,20]. There is also some thought that parental overprotection can inhibit social development, particularly in adolescence. Parental overprotection can result in parents limiting the child’s social activity opportunities due to their fears of organ rejection or infection [20-21]. Further, reseach has suggested that the negative side-effects the immuno-suppressants have on physical appearance may result in body image issues and low self-esteem, which can impact the recipient’s functioning in social situations [20]. In addition, the recipient’s own anxieties about their health, discomfort with being treated as a healthy individual, and fear of rejection by peers may prevent them from engaging in social activities [20]. 

### Self-Concept and Self-Esteem

There is a dearth of research examining self-esteem and self-concept in pediatric heart transplant recipients. Concerns have been raised regarding feelings of inadequacy or inferiority in heart transplant recipients secondary to impact of immunosuppresants on physical appearance, activity limitations, and cognitive impairment [5,11,20]. Further, there is some thought that just the awareness of being different can have a negative impact on self-esteem as acceptance by peers is one of the primary goals during adolesence. 

There have however, been no consistent research findings in this domain. Uzark *et al* (1992) found that heart transplant recipients assessed themselves similarly to their healthy peers on measures of self-concept and self-esteem [11]. In contrast, Mellander, Berntsson, and Nilsson (2006) found that children who had undergone the Norwood procedure as treatment for HLHS expressed more feelings of inferiority and lower self-esteem than their peers [22]. Other studies examining self-esteem and self-concept in heart transplant recipients and children/adolescents with congenital heart disease have noted that a significant percentage of the adolescents evidenced poor self-concept [23-24]. It is difficult to compare these studies as the methodology, including participants and measures, were considerably different in each study. However, the paucity of data examining these factors, inconsistent findings, and reasons for concern beg for additional research on this topic. 

### Behavioral and Emotional Functioning

Research on psychosocial functioning in pediatric transplant recipients indicate that the majority adapt to the transplantation without significant difficulty [13,25]. A significant minority, however, are noted to exhibit clinical levels of behavioral, emotional, and/or psychiatric difficulties. Studies have suggested that between 20 and 52% of the recipients exhibit behavioral issues, the majority consistent with depression [6,11,13-14,20,26]. Uzark *et al* (1992) noted that approximately one third of recipients in their study, assessed 4-60 months after transplantation, had behavioral difficulties [11]. Serrano-Ikkos *et al* (1999) found that over 50% of the heart transplant recipients experienced emotional and behavioral difficulties [14]. Wray and colleagues followed heart transplant recipients for three years following transplant and found again that approximately one third of the participants evidenced behavioral problems, when only those participants that completed all three evaluation sesisons were considered [6,15,20]. One point of interest in the Wray *et al* studies was that recipients were primarily exhibiting behavioral difficulties at home and not at school, as rates of behavioral difficulties at school significantly decreased over the three year period. 

In terms of psychiatric symptoms, Serrano-Ikkos *et al* (1999) found that 26% of the participants had a diagnosable psychiatric disorder consistent with Anxiety, Depression, and Adjustment disorder [14]. Wray *et al* (2004) noted depressive symptoms in approximately 13% of transplant recipients in their study [16]. Several studies also examined the psychosocial functioning of recipients before and after transplantation and have consistently found a decrease in psychiatric and behavioral symptoms from pretransplantation to posttransplantation [13-14,16,27]. 

Overall, a significant percentage of pediatric heart transplant recipients have consistently been found to have behavioral and psychiatric difficulties. One caveat to this discussion is that none of these studies have focused solely on adolescents, the majority consisting of convenience samples that include both children and adolescent recipients. 

### Quality of Life

Assessing quality of life is one method of study for addressing the effectiveness of a medical procedure, such as transplantation. There are many ways of assessing quality of life, but the most common in health-based research is to examine health-related quality of life (HRQoL), or the effect of medical ilnesses on everyday functioning. There is a significant paucity of objective data examining the HRQoL in pediatric heart transplant recipients as a majority of the researchers have utlized data regarding low incidence of medical consequences, school attendance, or reduction in behavioral and psychosocial functioning as evidence of improved HRQoL [28-30]. A single study examining HRQoL in a small sample of adolescent heart tranplant recipients noted average perceived HRQoL [31].

There have been a few studies assessing HRQoL in children with general cardiovascular conditions as well as with other pediatric transplant recipients [17-18,32]. One study examining the HRQoL of children and adolescents with cardiovascular disease found that recipients had significantly lower scores on all HRQOL domains in comparison to the normative population [32] DeMaso *et al* (2004) evaluated HRQoL in children and adolescents with Implantable Cardioverter-Defibrillators (ICDs) for correcting ventricular arrhythmias and noted that the participants had signifcantly lower physical quality of life [17]. The participants also obtained lower scores on multiple psychosocial quality of life domains including the social limitations and self-esteem. Additionally, the study noted that illness severity was not correlated with quality of life. Studies examining HRQoL in pediatric liver and renal transplant recipients also indicated lower scores on all domains of the HRQoL, similar to children with chronic medical condition [33-35]. Bucuvalas *et al* (2003) in evaluating pediatric liver transplant recipients noted that age at time of transplant, time since transplantation, and the number of hospitalizations the previous year were all predicitive of HRQoL [33]. 

### Adherence

Adherence is an important topic of investigation, particularly with adolescent transplant recipients, because of the negative impact it can have on their health status and mortality [36]. Adolescent transplant recipients appear to be at particular risk for non-adherence for multiple reasons. First, the adolescent time period itself appears to be a risk factor for non-adherence due to increased need during this period to conform with their peer group and suppress any qualities that make them appear different [19]. Additionally, body-image becomes very important during this period as it is associated with peer acceptance, and the negative impact immunosuppresant therapy has on physical appearance may cause adolescents, especially girls, to stop taking the medication [19]. Third, parents may expect adolescents to be more responsible for their own medical management and provide less supervision than they would with younger children [37]. Fourth, there is data from pediatric cancer and the adult transplant literature that suggests patients become less adherent to medical regimen over time, which connotes increased rates for adolescence given that many of them were transplanted as infants or younger children [38-39]. Finally, the normal stressors that occur during adolescence can interact with the stressors that are a result of the chronic illness to create psychological distress, which also increases the risk of non-adherence [25]. 

There are again very few studies examining non-adherence in pediatric transplantion recipients. Serrano-Ikkos *et al* (1998) examined adherence to Cyclosporine, through blood level assays, in child and adolescent heart transplant recipients and found that approximately one third of the participants presented with non-optimal adherence [37]. The study also noted that there was significant age difference as all of the participants who evidenced poor adherence were older children and adolescents, over the age of 10. Wray *et al* (2006) evaluated nonadherence to medication in adolescent heart transplant recipients through the use of questionnaires and noted 28% of the recipients engaged in unintentional non-adherence, “forgetting to take the medication”, and 18% engaged in delibrate non-adherence [40]. Unintentional non-adherence was associated with being preoccupied with the illness and its effects and concern about the effect of medication; while delibrate non-adherence was related to depressive symptoms and medical side-effects to the transplant procedure. Overall the two studies noted that a significant percentage of the recipients are non-adherent with the medical regimen, consistent with data obtained regarding non-adherence in other pediatric transplant recipients (liver and kidney) and those with chronic diseases [41-43].

### Literature Summary

There are significant limitations in the current understanding of the long-term psychosocial sequelae of pediatric heart transplantation. The low number of existing studies, the variability in the participants and methodologies make it difficult to generate conclusions based on this data. Further, studies in only one domain have actually focused on adolescence, non-adherence, with all others evaluating a sample of children and adolescents. Additionally, none of the studies have examined functioning beyond the 10 year mark and most assessed functioning 1-3 years after transplantation, thus any conclusions that can be drawn from this data is limited to this short outcome period. In the following section the authors present preliminary data from an ongoing study assessing psychosocial functioning and quality of life in adolescents who recived their heart transplant during infancy. 

## STUDY 1: METHODS

### Participants and Procedures

All infants who had undergone heart transplant at Loma Linda University Children’s Hospital were provided opportunities to participate in neurodevelopmental and psychosocial evaluations through the Department of Pediatrics Psychology Services as part of the LLU International Heart Institute’s standard of care and/or research programs via institutionally approved IRB studies. Only caregivers who have consented to their child’s information being utilized for research purposes are included. For the current study, the study investigators specifically identified adolescents between the ages of 12 and 17, who were eligible to participate in this study. The families of these identified adolescents were mailed a packet consisting of a letter explaining the study, consent forms, and psychosocial and quality of life questionnaires to be completed by the adolescent and a caregiver. The transplant team coordinator contacted the families to remind them to return the completed questionnaires 5-6 weeks after the packets were initially mailed out. The families contacted included both those who were obtaining follow-up care at LLUCH and at other centers; however, all of them had obtained their heart transplant at LLU Children’s Hospital.

The return rate for the packets at the time of analysis was approximately 34%. Participants included 22 adolescents (14 males, 8 females) between the ages of 12 and 17 years (Mean Age = 15yrs, SD = 1.6) and their caregivers. They were predominantly Caucasian (n = 14), followed by Latino (n = 7), and 1 individual of unspecified ethnicity. The participants were all transplanted prior to the age of 1 (Mean Age at time of transplant = 74.2 days, 87.7 SD). 

### Measures

#### Behavior Assessment System for Children, Second Edition (BASC-2):

The BASC-2 is a highly reliable and valid multimodal system for assessing behavior, psychosocial, and adaptive functioning in children and adolescents [44]. Two forms of the BASC-2 were administered to the participants in the study – the parent report form (BASC-2 PRS) and the adolescent self-report form (BASC-2 SRP). BASC-2 subscale and composite scores are T-scores with a mean of 50 and standard deviation of 10. Scores above 60 are considered to be in the clinical range for behavior scales and composite, while scores below 40 are considered to be in the clinical range for adaptive scales. 

BASC-2 PRS consists of 14 subscales which compose 4 composites assessing overall behavior problems. Subscales and composites of this measure were utilized to examine the self-esteem, social functioning, and behavioral functioning of adolescent heart transplant recipients. 

BASC-2 SRP consists of 16 subscales that derive 5 composite scores examining overall self-perception of behavioral functioning. Similar to the BASC-2 PRS, many but not all subscales and composites were utilized in this study in assessing adolescent self-perceptions of behavior, social functioning, and self-esteem and self-concept. 

#### Piers-Harris Self-Concept Scale, Second Edition (PH-2):

PH-2 is a self-report measure of self-esteem and self-concept. The measure assesses self-concept along 6 dimensions – Behavioral Adjustment, Intellectual and School Status, Physical Appearance and Attributes, Freedom from Anxiety, Popularity, and Happiness and Satisfaction. The composite and total scores are T-scores with a mean of 50 and standard deviation of 10, with scores below 40 indicating clinical concern. The measure has adequate reliability and is normed for children between the ages of 7 and 18 years [45]. 

#### Trauma Symptoms Checklist for Children (TSCC):

TSCC is a self-report measure of posttraumatic distress and psychiatric symptoms in children and adolescents between the ages of 8 and 16 years. The alternate version of the TSCC was used in this study and it is noted to assess for symptoms along 5 clinical dimensions – anxiety, depression, anger, posttraumatic stress disorder, and dissociation. The scores of all 5 scales are T-scores with mean of 50 and standard deviation of 10, with scores above 60 indicating clinical concern. The measure is noted to have acceptable reliability and validity for use with children and adolescents who have experienced a traumatic event [46]. 

#### Children’s Health Questionnaire, parent form (CHQ-PF50):

Parents and caregivers completed the CHQ-PF50 as a measure of the participants’ health-related quality of life. CHQ-PF50 assesses health, behavioral, and psychological functioning from 11 multi-item scales and 2 composites – Physical and Psychosocial Quality of Life. Higher scores on the scales indicate better functioning on those domains. The Physical and Psychosocial Quality of Life composite scores are T-scores with a mean of 50 and standard deviation of 10, with scores below 40 indicating poor quality of life. The measure is highly reliable and well validated for use with chronically ill children and adolescents [47]. 

### Analyses

Descriptive statistics were used to assess the behavioral, psychological, and social functioning of the participants as well their self-concept and quality of life. One-way analysis of variance was used to evaluate for differences in functioning by gender and ethnicity. Additionally, bivariate correlations were conducted to examine the relationships between age and the different behavioral, self-concept, and quality of life factors. 

## STUDY 1: RESULTS

### Social Skills 

Both parent and adolescent perceptions of the participants’ social skills were obtained through completion of the study questionnaires. Overall, the participants’ social skills were assessed to be in the Average range. Table **1** provides mean scores and standard deviations on the different social skills scales obtained. Importantly, however, upon closer examination and looking at frequency distributions on the different scales a significant minority of the group are noted to have social difficulties. Parent report measures indicated that between 20 and 24% of the participants evidenced deficits at the clinical level that require intervention. Parents reported concerns regarding basic social skills such as being polite (saying “please” and “thank you”) and complimenting and encouraging others with more advanced skills such as engaging in extracurricular activities. They also indicated worries about being isolated from peers either through active avoidance on participants’ end or not having the opportunity to engage with others due to physical or psychological limitations. The frequency was slightly lower on the self-report measures; however, 15-23% of the participants indicated that they were experiencing social difficulties at the clinical level. These participants reported that they have difficulty relating to their peers, have limited friends, and that social situations cause considerable stress and discomfort. 

One-way ANOVAs were conducted to determine whether there were any differences in social skills by ethnicity or gender. No statistically significant differences were noted by ethnicity (F = 0.104-2.496, p = 0.902-0.112) or by gender (F = 0.000-3.523, p = 1.00-0.076). Bivariate correlations were run to examine associations between age and social skills. Moderate positive correlations were noted between parent report of social skills and age, indicating that parents of the older participants perceive them to have better social skills than the parents of the younger participants. Moderate negative correlations were found between parent report of withdrawal behaviors and age, indicating younger participants tend to withdraw more than older participants.

### Self-Concept and Self-Esteem

Both parent and self-perceptions of the participants’ self-concept and self-esteem was obtained through multiple study questionnaires and scales. The self-concept and self-esteem of the overall group was found to be within normal limits (See Table **2**). A percentage of the group, however, did evidence significantly lower scores than the population mean. On a parent reported scale, roughly 30% of the participants obtained scores more than a standard deviation below the population mean. This scale included items assessing the parents’ perceptions of participants’ satisfaction with their school functioning, peer relationships, and life overall (CHQ-PF50 Self-Esteem). In addition, self-report measures indicated that between 5 and 25% of the participants reported clinical scores on various self-concept and self-esteem domains. Participants generally did not report concerns with their appearance, their intellectual and school status, or their behavior. They were, however, more likely to assess themselves as being inadequate in meeting their own or others’ expectations and in being less confident in their ability to make decisions and cope with difficulties they are likely to experience. Further, approximately 15-29% of the participants assessed themselves as having very low self-esteem. Overall, a significant percentage of the participant group reported problems with self-esteem and self-concept including feeling of inadequacy, lack of confidence in self, low ego strength, and poor peer relationships. 

No differences in self-concept by ethnicity or gender were noted (Ethnicity: F = 0.088-1.908, p = 0.916-0.181; Gender: F = 0.000-3.199, p = 1.00-0.089). One scale that did approach significance was Physical Appearance and Attributes on which girls had lower scores than boys (Female = 48.89 & Males = 56.38). This suggests girls report less satisfaction with appearance than boys, though both group means are within normal limits. Further, moderate negative correlations were noted between age and several self-concept domains including BASC-SRP Self-Reliance (r = -0.321) and Physical Appearance and Attributes (r = -0.235), indicating that older adolescents reported more problems with their self-confidence and appearance than younger children (pre-adolescents).

### Behavior

Similar to the outcomes in the other areas measured in this study the participants’ behavior overall was assessed to be in the Average range on both parent and self-reports, though a significant percentage were noted to have clinical scores on various behavioral scales (See Table **3**). Approximately 40% of the participants were assessed to be in the clinical range on the overall behavior scale based on parent report in comparison to 25% by self-report. Concerns were predominantly raised on attention regulation (29% parent, 30% adolescent), depression (29% parent, 12-15% adolescent), hyperactivity (24% parent, 15% adolescent), atypicality (25% parent, 5% adolescent), and anxiety (14% parent, 12-20% adolescent). Further, parents reported that a significant percentage of the adolescents engage in withdrawal behaviors (24%); while adolescents indicated that many feel they have little control over their own lives (25%). A significant percentage of the participants were noted to have physical ailments or limitations that are impacting their functioning (43% parent, 30% adolescent)¸ however, given the participants’ medical issues, this should not be interpreted beyond noting that the participants’ do have limitations that are impacting their functioning. 

There were no differences in behavior by gender or ethnicity. Moderate positive and negative correlations were noted between age and different behavioral scales. Positive correlations were noted between age and self-report attention problems (r = 0.361), self-report anxiety (PH-2 Freedom from Anxiety r = 0.388), and parent report measure of behavior (CHQ-PF50 r = 0.237). Negative correlations were found between age and parent-report withdrawal behaviors (r = -0.306) and self-report need for risky or sensation seeking behaviors (r = -0.301). This suggests that older adolescents evidence more attention problems but less withdrawal behaviors, sensation seeking behaviors, and anxiety than younger adolescents. The behavior of older participants is also rated more positively than behaviors of younger participants. 

### Quality of Life

Parents overall assessed participants’ health related quality of life to be within normal limits both on the physical and psychosocial domains (see Table **4**). Subscale means were also roughly close to the population means on the CHQ-PF50 except on the General Health Perceptions scale, which was over a standard deviation below the population mean (z = -1.243). In terms of frequency, a majority of the parents expressed concerns about participants’ general health, with over 75% indicating their child’s current health is poor and will continue to deteriorate, and presence of pain and discomfort (25%). They also indicated that the participant’s health considerably lessens their social and day-to-day functioning (20%). Further, a significant number of parents indicated that their child’s health has an impact on their own emotional functioning and consumes much of their time (Parental Impact Emotional = 25%; Parental Impact Time = 20%). As such, a considerable percentage of the participant pool acknowledged functional limitations to physical ailments and poor physical quality of life. Most parents assessed their children’s psychosocial quality of life to be average or above average, though a percentage did indicate concerns regarding their child’s self-esteem. Approximately 20% of the participants were also noted to be limited in their social interactions because of their behavior or emotional functioning. Overall, participants’ psychosocial quality of life was assessed to be significantly superior to their physical quality of life, though both were primarily within normal limits. 

No differences in quality of life were noted by either gender or ethnicity (F = 0.000-2.749, p = 1.00-0.092). One scale that did approach significance was CHQ-PF50 Bodily Discomfort (F = 2.749, p = 0.092), with Caucasian participants obtaining lower rating on pain level and frequency than Latino participants (Caucasian = 81.54, Latino = 65.00). Moderate positive correlations were noted on several of the psychosocial scales (Psychosocial Summary = 0.363 and Behavior = 0.237) and parental impact scales (Parental Impact Emotional = 0.544 and Parental Impact Time = 0.566). 

## STUDY 1: DISCUSSION

The results of this preliminary examination are in many ways consistent with the existing literature on the behavioral and psychosocial functioning of heart transplant recipients. DeMaso *et al* (2009) indicated that a majority of children and adolescents with chronic illnesses do not present with noticeable emotional or behavioral difficulties, but may be at risk for subclinical levels [18]. Similarly, a majority of the participants in this study did not evidence significant behavioral, emotional, social, or physical ailments. They are functioning equivalently to their peers, with the exception of a greater focus on their health. A significant minority, however, do present with clinical difficulties that should be clinically addressed. 

Approximately 24% of the participants presented with social deficits including impairments in basic social functioning, avoidance of social engagement, and discomfort with social situations. This is slightly lower than rates in previous studies by Uzark *et al* (1992) and Wray and Radley-Smith (2006) [11,20]. Further, no gender differences were noted with regard to social functioning, contradicting previous findings suggesting boys exhibited greater deficits than girls^14^. The lower rate in this study could be attributed to the demographics of the participants group, as they were on the whole significantly older than either of those participant groups and consisted of fewer girls. Further, it could be hypothesized that transplant recipients acquire more appropriate social skills as they developmentally mature into adolescence and spend less time in hospitals or have had longer adaptation time to their functional status. The greater biological and cultural impetus to cultivate peer social relationships during adolescence may also be positively influencing recipients to develop social skills and engage in social interactions. This may be particularly true for adolescent male recipients, as there appears to be more cultural focus on adolescent males becoming independent from parents and family than adolescent females [48-49]. 

A significant percentage of the study participants were also noted to have poor self-esteem and self-concept, particularly in relation to feeling inadequate in meeting expectations, making decisions, and feeling a lack of control over life events. The data to date examining self-esteem in pediatric transplant recipients has been inconclusive with several studies indicating that they have lower self-esteem and other stating that there is no difference between their self-esteem and that of healthy controls [11,22-24]. Further, in studies that have suggested that they have lower self-esteem, the causes for the lower self-esteem has varied ranging from activity restrictions due to neurological or physical problems to differences in physical appearance. Adolescents in this study indicated that they did not feel different from their peers on physical appearance, intellectual or school functioning, and experienced limited activity limitations. Their primary symptoms were feelings of ineffectiveness and inadequacy. Given that the participants in the previous studies were considerably younger; it may be that these study participants are more cognitively aware of their functioning differences and as such the difficulties they are encountering. Additionally, adolescents are likely more focused on the future, their potential and expectations placed on them, than children; this likely reflects in the type of symptoms they endorsed.

Research acknowledging behavioral difficulties and psychiatric symptoms in pediatric heart transplant recipients is quite common. The majority of the studies have indicated that between 20 and 50% of the participants exhibit some behavioral or emotional symptoms, mainly internalizing problems such as depression. The results of this study are consistent with previous studies stating that about 40% of participants presented with significant behavioral or emotional problems. The primary problem for this age group, in contrast to previous study groups, was attention problems. A significant percentage were also noted to exhibit depressive symptoms similar to previous studies, however the high prevalence rate of attention problems was a new finding unique to this study. These results may be limited to this study’s population, the higher number of male gender participants, or behavior problem areas may change over time. Depressive and anxious symptoms can also cause adolescents to appear inattentive and highly distracted thus suggesting the need for more in-depth understanding of this endorsement. Further, heart transplant recipients are noted to present with cognitive and language weaknesses, which may be contributing to the attention difficulties [50] and are a symptom of their other neuropsychological concerns rather than purely an ‘attention’ deficit. It may be that these symptoms are exaggerated during adolescence because of the stressors and demands placed on them during this period. 

HRQoL data from this study suggests that transplant recipients’ overall quality of life is similar to that of their healthy peers, though there are more concerns regarding their health and they present with higher rates of physical discomfort. DeMaso *et al* (2004) obtained similar results when examining quality of life in children with ventricular arrhythmias [17]. Overall, the results indicate that adolescent recipients present with mild impacts to their physical functioning but generally appropriate psychosocial functioning.

## STUDY 1: CONCLUSIONS

During adolescence, a majority of infant heart transplant recipients are functioning similarly to their peers in terms of behavioral, emotional, and social health. Their quality of life is also generally high, though there are concerns about their health and bodily discomfort. A small but significant percentage, however, were noted to have social deficits, low self-esteem, and behavioral difficulties related to attention problems and internalizing symptoms such as depression and anxiety. Given that adolescence is period of tremendous growth and change, there are concerns about functional status of this small group and their ability to acquire the different life skills inherent to this period. 

## STUDY 1: LIMITATIONS & FUTURE DIRECTIONS

This current study is merely a snap-shot of functioning during adolescence. It is difficult to draw conclusions based on this data on whether functioning has improved over time for heart transplant recipients. There is also no way to draw conclusions regarding whether the recipients’ current functioning is directly related to their medical issues. This data does, however, inform us on current functional status and offers suggestions as to what issues pediatric heart transplant recipients may be at risk for and hence allow for more targeted intervention. 

The psychosocial development of infant heart transplant recipients should be examined longitudinally to determine the relationship between medical issues and functioning. Additionally, studies need to begin examining the impact of recipient’s relationship with their parents on their functioning. 

## STUDY 2: NEURODEVELOPMENTAL FUNCTIONING IN PEDIATRIC HEART TRANSPLANT RECIPIENTS

Severe cardiac defects and diseases requiring transplantation are noted to impact neuropsychological and cognitive development [6,50]. This section provides an overview of the literature discussing neurodevelopmental outcomes in pediatric heart transplant recipients AND then includes related preliminary data from adolescents who received their heart transplant during infancy from the authors' ongoing research studies in this area. 

### Neurodevelopmental Outcomes 

Cognitive and neuropsychological functioning remain areas of concerns in child and adolescent heart transplant recipients. Consistently research to date has demonstrated that recipients evidence delays in development and lower cognitive functioning in relationship to healthy peers [6-9,50-51]. Studies evaluating early development in infant recipients have noted low average to average performance on the mental and motor indices [8,50-51]. These studies also indicated that a significant percentage of the participants were well below the mean on both indices. Further, one longtitudinal study demonstated a decrease in mental index scores over time, likely the result of the predominance of langauge and abstract reasoning tasks at the older ages [51]. 

In older heart transplant recipients, cognitive functioning was found to be in the Average range but signficantly lower than that of healthy peers [6-9,50,52-53]. Impairments in receptive and expressive language were noted as well, with participants generally performing 0.66 to 1 standard deviation below the mean [50]. Further, Baum *et al* (2004) noted that academic functioning was consistent with the recipients’ cognitive potential, but significantly lower than that of the normative sample [7]. The study also noted that the recipients had more difficulty with spelling, arithmetic, and language skills; and that a significant percentage of the participants had learning difficulties and needed special education services. 

### Literature Summary 

Overall, the existing literature suggests that transplant recipients present with impairments in cognitive, academic, and neuropsychological functioning. While more information is available regarding pediatric transplant recipient’s neurodevelopmental functioning, there are still limitations to the current understanding as few of the studies examined functioning beyond childhood.. The following presents neurodevelopment data from ongoing studies at LLUCH in regards to adolescent infant heart transplant recipients. 

### Study 2: Methods

#### Participants and Procedures

Infants who had undergone heart transplant at LLUCH were provided opportunities to participate in neurodevelopmental and psychosocial evaluations through the Department of Pediatrics Psychology Services as part of LLU International Heart Institute’s standard of care. The evaluation protocol includes assessment of cognitive, academic, and neuropsychological functioning. Parents provide consent to use data collected clinically for research purposes at the time of evaluation. Data collection was approved by the Institutional Review Board and informed consent from the parent and, when appropriate, assent from the child was obtained.

Cognitive, academic, and neuropsychological from all infant heart transplant recipients who were between the ages of 12 and 17 at time of evaluation were utilized in this study. This included 21 participants (6 females, 15 males) between the ages of 12 and 16 (Mean Age = 13.7, 1.1SD), all of whom were transplanted prior to the age of 1 (Mean Age at time of Transplant = 42.43 days, 47.3SD). The ethnic composition of the group was predominantly Caucasian (n = 16) followed by Latino (n = 4) and African American (n = 1). 

#### Measures 

##### Wechsler Abbreviated Intelligence Scale (WASI):

WASI is a brief but reliable measure for screening intelligence in individuals between the ages of 6 and 89. It is composed of 4 subtests (Vocabulary, Similarities, Matrix Reasoning, and Block Design) and yields 3 composite scores Full Scale Intelligence Quotient (FSIQ), Verbal Intelligence Quotient (VIQ), and Performance Intelligence Quotient (PIQ). Subtest scores are T-scores with a mean of 50 and a standard deviation of 10, while composite scores are standard scores with a mean of 100 and a standard deviation of 15. WASI subtests assess the same constructs as their counterparts on the full Wechsler Intelligence Scale for Children (WISC) and Wechsler Adult Intelligence Scale (WAIS), and correlations between WASI and the WISC/WAIS for the composite scores ranged between 0.76 and 0.92, indicating that is valid measure of cognitive functioning [54]. 

##### Wechsler Individual Achievement Test - Second Edition Abbreviated (WIAT-II-A):

WIAT-II-A is an abbreviated measure of academic achievement in individuals between the ages of 6 and 85. It consists of 3 subtests – Basic Reading, Mathematics Reasoning, and Spelling; which were derived from the full WIAT-II. Subtest scores are standard scores with a mean of 100 and standard deviation of 15. Correlations between the WIAT-II-A and WIAT-II are fairly highly, indicating that WIAT-II-A is a valid measure of academic achievement [55]. 

##### Clinical Evaluation of Language Fundamentals, Fourth Edition (CELF-4):

CELF-4 is a reliable and valid measure of language functioning in children and adolescents between the ages of 5 and 16. Only the subtests that compose the Core Language, Receptive Language, and Expressive Language composites were administered to the participants. Composite scores are standard scores with a mean of 100 and standard deviation of 15 [56]. 

##### The Beery-Buketenica Developmental Test of Visual-Motor Integration (VMI):

VMI assesses the ability of children and adolescents, between the ages of 2 and 18, to integrate their visual and motor skills in copying geometric designs of increasing difficulty. Scores are standard scores with a mean of 100 and standard deviation of 15. VMI is considered to be one of the most researched and a validated measure of visual motor integration [57]. 

##### Behavior Rating of Executive Function, Parent Form (BRIEF):

BRIEF is a parent report of executive functioning behaviors of children and adolescents between the ages of 5 and 18. It yields 3 composite scores that assess overall executive functioning, behavioral and emotional inhibition and regulation, and higher-order cognitive processes such as working memory, planning and organizing, initiating tasks, and monitoring progress. Composite scores are T-scores with mean of 50 and standard deviation of 10. BRIEF is a reliable and valid measure of executive functioning behaviors [58]. 

#### Analyses

Descriptive statistics were used to assess the cognitive, academic, and neuropsychological functioning of the participants. One-way analysis of variance was used to evaluate for differences in functioning by gender and ethnicity. Additionally, bivariate correlations were conducted to examine the relationships between age and cognitive, academic, and neuropsychological factors. 

### Study 2: Results

#### Cognitive Functioning

Participants cognitive functioning overall was noted to be in the Below Average range (FSIQ = 80.14, 18.0SD; VIQ = 80.71, 18.7SD; PIQ = 82.71, 16.7SD). A significant percentage of the participants, however, were functioning at well below average levels on the different domains – 53% were in the Borderline to Impaired range on FSIQ (below 80), 62% on VIQ, and 43% on PIQ. Further, a very small percentage of recipients were functioning within normal limits or above, particularly on FSIQ and VIQ (24%). These scores suggest that majority of the participants are cognitively functioning at levels significantly below expected.

One-way ANOVAs were conducted to determine whether there are any differences in cognitive functioning by gender or ethnicity, and none were noted (Ethnicity: F = 0.389-2.212, p = 0.684-0.138; Gender: F = 0.058-0.662, p = 0.812-0.514). Moderate correlations were noted between age and the cognitive scales (FSIQ r = 0.206, VIQ r = 0.208, PIQ r = 0.267), indicating that older participants obtained slightly higher scores than younger participants. 

#### Academic Functioning

Participants overall obtained low average to average scores on the academic screener- Word Reading = 87.29 (20.4SD), Numerical Operations = 82.45 (26.0SD), Spelling = 90.90 (20.8SD). Additionally, 30-45% participants performed in the Borderline to Impaired range on the academic scales, indicating that a significant percentage of the group do present with academic difficulties. There was no difference in academic scores by gender or ethnicity, and correlations between age and academic scales were very small (r = - 0.019-0.039). 

#### Neuropsychological Functioning

Participant’s language, visual-motor integration, fine motor, and executive functioning skills were assessed as part of their neuropsychological functioning. Participants demonstrated significant difficulty with language skills, performing overall in the Borderline range (Overall Language = 78.93, 19.8SD; Receptive Language = 77.54, 18.3SD; Expressive Language 74.31, 18.8). Further, 53-61% of the participants performed below expected standards on the language skills. The visual-motor integration ability of the group was noted to be below average (VMI M = 81. 36, 10.8SD), with approximately 27% functioning in the impaired range. The majority of the participants (71-86%) also demonstrated significantly below average fine motor control and coordination. Finally, 19-25% of the participants were reported to have executive functioning deficits, particularly with working memory, planning, and organization. Overall, a large percentage of the participants evidenced significant deficits in their neuropsychological functioning. 

## STUDY 2: DISCUSSION

Existing literature suggests that infant heart transplant recipients are generally in the Low Average range in terms of their IQ scores. Baum *et al* (2000) indicated that pre-school age children obtained mean IQ scores in the 70s, while school age children were in the 80s [53]. Malhe *et al* (2006) assessed school age children and determined their mean IQ scores to be in the 80s as well [9]. The current study examining cognitive functioning in adolescence similarly found mean IQ scores to be in the low 80s, suggesting that cognitive functioning is consistent across the different age groups of transplant recipients. Further, a large percentage of participants were functioning at levels well below average, raising concerns about their functional potential as they move into adulthood. 

Academic achievement for the overall group was low average, scores similar to that noted by Baum *et al* (2004) and Malhe *et al* (2006) [7,9]. Academic scores were, in fact, significantly higher than expected for the overall group based on their cognitive scores. This suggests that the recipients’ ability to learn is intact and that they are working hard to acquire information being taught to them. 

Performance in other neurodevelopmental domains was also consistent with what is noted in the existing literature and similar to other groups of pediatric populations who have had anoxic events (e.g. long-term outcomes of the premature child). Language functioning (both receptive and expressive) was borderline impaired, with over 50% of the participants functioning at levels significantly below average. Visual-motor integration skills of the group were low average as well. Finally, a significant percentage of the participants were reported to have executive functioning deficits, particularly with higher order skills such as planning/organization and initiating tasks, on parent report. There have been no previous studies examining executive functioning skills in heart transplant recipient, and these results suggest that it needs to be examined in greater depth. 

## STUDY 2: CONCLUSIONS

Adolescent heart transplant recipients are noted to be functioning in the borderline impaired to low average level on the cognitive and neurodevelopmental domains assessed. As mentioned, this is consistent with previous literature on younger preschool and school age children, indicating functioning is similar across all age groups. As there have been few longitudinal studies examining functioning, no firm conclusions can be drawn regarding whether functioning is stable over time, however, the various studies with different age samples would suggest this. 

## STUDY 2: LIMITATIONS AND FUTURE DIRECTIONS

The primary limitations of the study are the small participant pool and administration of abbreviated instruments or parent report forms rather than more comprehensive measures. This limits the data and the conclusions that can be generated. As such, more thorough evaluations using comprehensive measures should be utilized in the future. Longitudinal assessment will also beneficial as it will provide profiles of performance over time and allows conclusions to be drawn regarding the neurodevelopmental trajectories of the infant recipients. 

## FINANCIAL DISCLOSURE & CONFLICT OF INTEREST

The Authors have no financial interests or other conflicts of interest to disclose in regards to the contents of this article

## Figures and Tables

**Table 1:** Group Mean and Standard Deviations for the Social Skills Scales.

	Mean	Standard Deviation
BASC-2 PRS Social Skills^a^	50.67	8.9
BASC-2 PRS Leadership^a^	46.52	10.8
BASC-2 PRS Withdrawal^b^	52.57	9.2
BASC-2 SRP Interpersonal Skills^a^	51.15	12.6
BASC-2 SRP Social Stress^b^	48.30	16.1
PH-2 Popularity^a^	48.45	13.9
CHQ-PF50 Role/Social Limitation Emotional/Behavioral^c^	87.2	25.6
CHQ-PF50 Role/Social Limitation Physical^d^	91.7	15.8

Subscales from Behavior Assessment System for Children, Second Edition Parent and Self-report forms (BASC-2 PRS & SRP); Piers-Harris, Second Edition (PH-2); and Children’s Health Questionnaire Parent Form (CHQ-PF50) were utilized to assess social functioning.

a Subscales have a mean 50 and standard deviation of 10, with scores below 40 indicating clinical concern.

b Subscales have a mean 50 and standard deviation of 10, with scores above 60 indicate clinical concern.

c CHQ-PF50 Role/Social Limitation Emotional/Behavioral normative population mean = 90.4, 19.5SD

d CHQ--PF50 Role/Social Limitation Physical normative population mean = 91.5, 18.9SD

**Table 2:** Group Means and Standard Deviations for the Self-Concept Scales

	Mean	Standard Deviation
CHQ-PF50 Self-Esteem^a^	74.78	16.6
BASC-2 SRP Sense of Inadequacy^b^	53.20	12.9
BASC-2 SRP Self-Esteem^c^	49.75	12.2
BASC-2 SRP Self-Reliance^c^	47.80	10.8
PH-2 Behavioral Adjustment^c^	52.36	10.2
PH-2 Intellectual and School Status^c^	47.77	9.5
PH-2 Physical Appearance^c^	53.32	10.2
PH-2 Freedom from Anxiety^c^	49.36	11.9
PH-2 Popularity^c^	48.45	13.9
PH-2 Happiness and Satisfaction^c^	51.23	8.8
PH-2 Total^c^	51.59	12.3

Subscales from Behavior Assessment System for Children, Second Edition Self-report form (BASC-2 SRP); Piers-Harris, Second Edition (PH-2); and Children’s Health Questionnaire Parent Form (CHQ-PF50) were utilized to assess self-concept and self-esteem functioning.

a CHQ-PF50 Self-Esteem normative population mean = 79.3, 17.8SD

b Subscales have a mean 50 and standard deviation of 10, with scores above 60 indicating clinical concern

c Subscales have a mean 50 and standard deviation of 10, with scores below 40 indicating clinical concern

**Table 3:** Group Means and Standard Deviations for the Behavior Scales

	Mean	Standard Deviation
BASC-2 PRS Behavioral Symptoms Index^a^	53.30	10.8
BASC-2 PRS Externalizing Behavior^a^	49.50	7.0
BASC-2 PRS Hyperactivity^a^	51.62	9.0
BASC-2 PRS Aggression^a^	48.05	7.7
BASC-2 PRS Conduct Problems a	49.00	6.8
BASC-2 PRS Internalizing Behavior^a^	55.00	11.1
BASC-2 PRS Anxiety^a^	49.05	9.7
BASC-2 PRS Depression^a^	55.05	10.6
BASC-2 PRS Somatization^a^	58.05	12.1
BASC-2 PRS Atypicality^a^	55.25	13.3
BASC-2 PRS Withdrawal^a^	52.57	9.2
BASC-2 PRS Attention Problems^a^	52.95	10.7
BASC-2 SRP Emotional Symptoms Index^a^	50.50	13.8
BASC-2 SRP Internalizing Problems a	51.45	13.6
BASC-2 SRP Atypicality^a^	51.10	11.6
BASC-2 SRP Locus of Control^a^	50.40	12.2
BASC-2 SRP Social Stress^a^	48.30	16.1
BASC-2 SRP Anxiety^a^	49.50	12.8
BASC-2 SRP Depression^a^	48.55	12.1
BASC-2 SRP Sense of Inadequacy^a^	53.20	12.9
BASC-2 SRP Somatization^a^	54.40	11.5
BASC-2 SRP Inattention/Hyperactivity^a^	50.70	11.5
BASC-2 SRP Attention Problems^a^	52.65	13.0
BASC-2 SRP Hyperactivity^a^	48.55	9.2
BASC-2 SRP Sensation Seeking^a^	47.45	10.6
PH-2 Freedom from Anxiety^b^	49.36	11.9
TSCC Anxiety^a^	48.38	8.6
TSCC Depression^a^	46.75	8.6
TSCC Anger^a^	43.33	6.5
TSCC PTSD^a^	47.42	10.1
TSCC Dissociation^a^	49.71	9.5
CHQ-PF50 Behavior	78.47	15.1
CHQ-PF50 Mental Health^c^	81.25	12.3

Subscales from Behavior Assessment System for Children, Second Edition Parent and Self-report forms (BASC-2 PRS & SRP); Piers-Harris, Second Edition (PH-2); Trauma Symptom Checklist for Children (TSCC); and Children’s Health Questionnaire Parent Form (CHQ-PF50) were utilized to assess behavioral functioning.

a Subscales have mean 50 and standard deviation of 10, with scores above 60 indicating clinical concern

b Subscales have mean 50 and standard deviation of 10, with scores below 40 indicating clinical concern

**Table 4:** Normative and Group Means and Standard Deviations for health related quality of life (HRQoL)

	Normative Mean (Standard Deviation	Group Mean (Standard Deviation)
Physical	50 (10)	45.78 (7.9)
Psychosocial	50 (10)	50.71 (10.5)
Physical Functioning	90.85 (16.4)	91.66 (14.1)
Social Limitation Emotional/Behavioral	90.40 (19.5)	87.22 (25.6)
Social Limitation Physical	91.50 (18.9)	91.67 (15.8)
Bodily Pain	78.68 (20.7)	77.50 (18.9)
Behavior	72.31 (17.1)	78.47 (15.1)
Mental Health	77.26 (13.7)	81.25 (12.3)
Self-esteem	79.26 (17.8)	74.78 (16.6)
General Health Perceptions	66.70 (19.4)	42.63 (22.0)
Parental Impact Emotional	73.98 (21.4)	62.08 (29.7)
Parental Impact Time	83.88 (20.3)	87.78 (16.5)

CHQ-PF50 subscales and composites scores were used to assess health related quality of life. This table presents the normative mean and standard deviations as well as the mean and standard deviation for the study participants
